# SMARCA4-deficient undifferentiated malignant tumor of the urinary bladder without a detectable coding SMARCA4 alteration: a case report and literature review

**DOI:** 10.3389/fonc.2026.1978166

**Published:** 2026-10-20

**Authors:** Yan Xue, Zhenyu Jin, Yihao Du, Zihang Huang, Zhengguo Zhang

**Affiliations:** Department of Urology, The First Affiliated Hospital of Zhengzhou University, Zhengzhou, China

**Keywords:** BRG1, disitamab vedotin, epigenetic silencing, immunohistochemistry, SMARCA4-deficient bladder tumor, tislelizumab

## Abstract

**Background:**

*SMARCA4* encodes BRG1, the ATPase catalytic subunit of the SWI/SNF chromatin remodeling complex. SMARCA4-deficient tumors are typically undifferentiated or rhabdoid in morphology, are aggressive, prone to early recurrence and are frequently resistant to conventional chemotherapy. In the bladder, such tumors are exceedingly rare, with only four cases reported to date, and no standard treatment has been defined.

**Case presentation:**

A 69-year-old man presented with painless gross hematuria and was found to have a pT3N0 tumor arising within a right bladder-wall diverticulum. Robot-assisted partial cystectomy yielded an undifferentiated malignant tumor with epithelioid/rhabdoid morphology, complete loss of BRG1 (SMARCA4) expression, HER2 expression (IHC 2+), low PD-L1 expression (tumor cell score(TC) <25%, immune cell population score(ICP) <1%) and a Ki-67 index of approximately 60%. Paired tumor and adjacent non-tumor whole-exome sequencing detected no detectable pathogenic coding *SMARCA4* point mutation or indel; copy-number and B-allele-frequency analyses further excluded deletion or loss of heterozygosity at 19p13.2, indicating that BRG1 loss in this tumor was not genomically encoded and was most plausibly epigenetically mediated. The patient received two cycles of adjuvant tislelizumab monotherapy. Intravesical recurrence occurred at 23 months and was treated with disitamab vedotin, a HER2-directed antibody-drug conjugate, combined with tislelizumab, followed by maximal transurethral resection and, at the time of bone progression, selective arterial embolization. Despite incomplete adherence to therapy, he remains alive 48 months after diagnosis, the longest survival reported for this entity to date.

**Conclusion:**

Immunohistochemistry, rather than sequencing, should be regarded as the diagnostic reference standard for *SMARCA4* deficiency, since BRG1 loss may occur without any detectable genomic alteration. Immune-checkpoint blockade combined with a HER2-directed antibody-drug conjugate produced meaningful and durable disease control despite low PD-L1 expression, and warrants prospective evaluation in this rare subtype.

## 1 Introduction

Urinary bladder cancer, a prevalent urologic malignancy, comprises no fewer than 40 histological subtypes, the majority of which demonstrate aggressive behavior and unfavorable clinical outcomes (1). Among these, SMARCA4-deficient bladder tumors constitute a distinct molecular subtype defined by *SMARCA4* inactivation and typically present with sarcomatoid or undifferentiated histology alongside markedly elevated proliferative indices (2).

At the molecular level, *SMARCA4* encodes BRG1, the ATPase catalytic subunit of the SWI/SNF complex, which participates in transcriptional regulation, DNA repair, and tumorigenesis. Somatic alterations of *SMARCA4* occur in 5%–7% of all malignancies, and only a small fraction of these arise in the bladder (3). Diagnostically, *SMARCA4*-deficient tumors can be identified by immunohistochemistry (IHC) or genetic testing; however, a substantial proportion of tumors with complete BRG1 loss carry no detectable *SMARCA4* mutation or deletion, which has been attributed either to epigenetic dysregulation (4) or to the limitations of current detection techniques (5).

Beyond these diagnostic challenges, *SMARCA4*-deficient tumors are clinically aggressive, with a marked propensity for recurrence even following complete surgical resection (6). No standard treatment regimen has been established; chemotherapy remains the conventional option, but responses are usually short-lived (7). To date, only four cases of primary *SMARCA4*-deficient bladder tumor have been reported, and only two of these were characterized at the genomic level (2, 8–10). Herein, we report an additional case in which complete BRG1 loss was demonstrated by IHC in the absence of any *SMARCA4* mutation, copy-number change, or loss of heterozygosity, and in which prolonged survival was achieved with combined immune-checkpoint blockade and HER2-directed antibody-drug conjugate therapy.

## 2 Case presentation

In July 2022, a 69-year-old man presented with painless gross hematuria of 2-month duration. He had a 30-pack-year smoking history and hypertension managed with amlodipine, but no diabetes, cardiovascular disease, prior malignancy, or relevant family history. Physical examination was unremarkable.

Contrast-enhanced pelvic MRI (**Figures 1A–C**) confirmed a solid-cystic mass in the right bladder region. CT urography (**Figures 1D, E**) revealed a cystic protrusion on the right bladder wall with soft tissue-density components and marginal calcifications, suggestive of a tumor arising within a right bladder diverticulum. Cystoscopy identified a broad-based, cauliflower-like mass within the diverticulum on the right lateral wall, with no other lesions in the bladder. Biopsy confirmed carcinoma. Chest CT (**Figure 1F**) showed no significant abnormalities, supporting a primary bladder origin. The patient underwent Da Vinci robot-assisted partial cystectomy with diverticulectomy and bilateral pelvic lymph node sampling.

**Figure 1:**
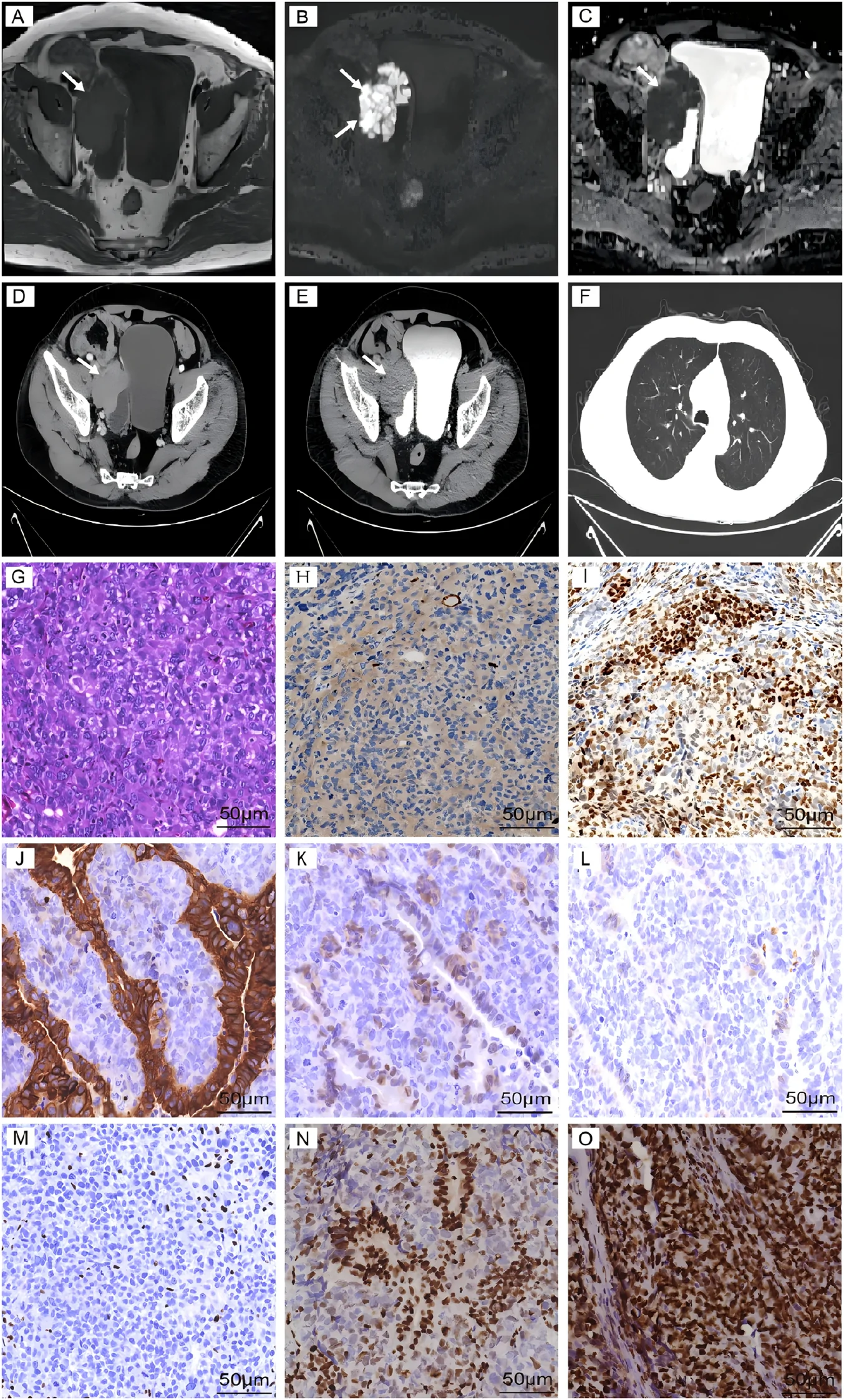
Radiological, histopathological, and immunohistochemical findings of a SMARCA4-deficient undifferentiated malignant tumor of the urinary bladder. Upper panel **(A–F)**: Radiological images obtained before tumor excision. **(A–C)** Gd-enhanced MRI images. **(D, E)** Multiphase-enhanced CT images. White arrows indicate the solid component of the tumor. **(F)** Baseline chest CT showing no significant abnormalities. Lower panel **(G–O)**: Histopathological and immunohistochemical findings. **(G)** H&E staining showing that the solid tumor consisted of undifferentiated tumor cells with pleomorphism, coarse chromatin, prominent nucleoli, frequent mitoses, and a dense lymphocytic infiltrate. **(H)** CD34 showing diffuse membranous/cytoplasmic positivity in tumor cells, with vascular endothelial cells as internal positive controls. **(I)** SOX2 showing strong and diffuse nuclear positivity. **(J)** CK7 showing focal and partial cytoplasmic positivity. **(K)** P63 showing focal and partial nuclear positivity. **(L)** CK20 showing focal and weak cytoplasmic positivity. **(M)** SMARCA4 (BRG1) showing loss of nuclear expression in tumor cells, with retained expression in stromal and inflammatory cells as internal control. **(N)** SMARCA2 (BRM) showing retained nuclear expression. **(O)** SMARCB1 (INI1) showing retained nuclear expression. Scale bars, 50 μm. HE, hematoxylin and eosin; MRI, magnetic resonance imaging; CT, computed tomography; Gd, gadolinium.

Postoperative pathology revealed an undifferentiated malignant tumor composed of large, discohesive cells with pleomorphism, coarse chromatin, prominent nucleoli, frequent mitoses, and a dense lymphocytic infiltrate (**Figure 1G**). Immunohistochemically, the tumor cells showed focal and partial expression of CK7 (**Figure 1J**) and P63 (**Figure 1K**), with focal weak expression of CK20 (**Figure 1L**), supporting the epithelial lineage. SMARCA4 (BRG1) expression was completely lost in tumor cells, with retained expression in stromal and inflammatory cells serving as internal control (**Figure 1M**). In addition, SMARCA2 (BRM) and SMARCB1 (INI1) nuclear expression was retained (**Figures 1N, O**), CD34 showed diffuse membranous/cytoplasmic positivity (**Figure 1H**), and SOX2 showed strong and diffuse nuclear positivity (**Figure 1I**). Ki-67 was ~60% in hot spots. PD-L1 was low (tumor cell score (TC) <25%, immune cell population score (ICP) <1%). The integrated diagnosis was SMARCA4-deficient undifferentiated malignant tumor of the urinary bladder, pT3N0, arising within a diverticulum.

Whole-exome sequencing of paired tumor and adjacent non-tumor tissue detected no pathogenic *SMARCA4* point mutations or indels (**Supplementary Figure 1**). Copy-number variation (CNV) and loss of heterozygosity (LOH) analyses further excluded deletion or LOH at 19p13.2 (**Figures 2A, B**), indicating that BRG1 loss was not genomically encoded. Promoter hypermethylation or other non-genomic mechanisms are the most plausible explanations, although promoter methylation and transcript-level analyses were not performed.

**Figure 2:**
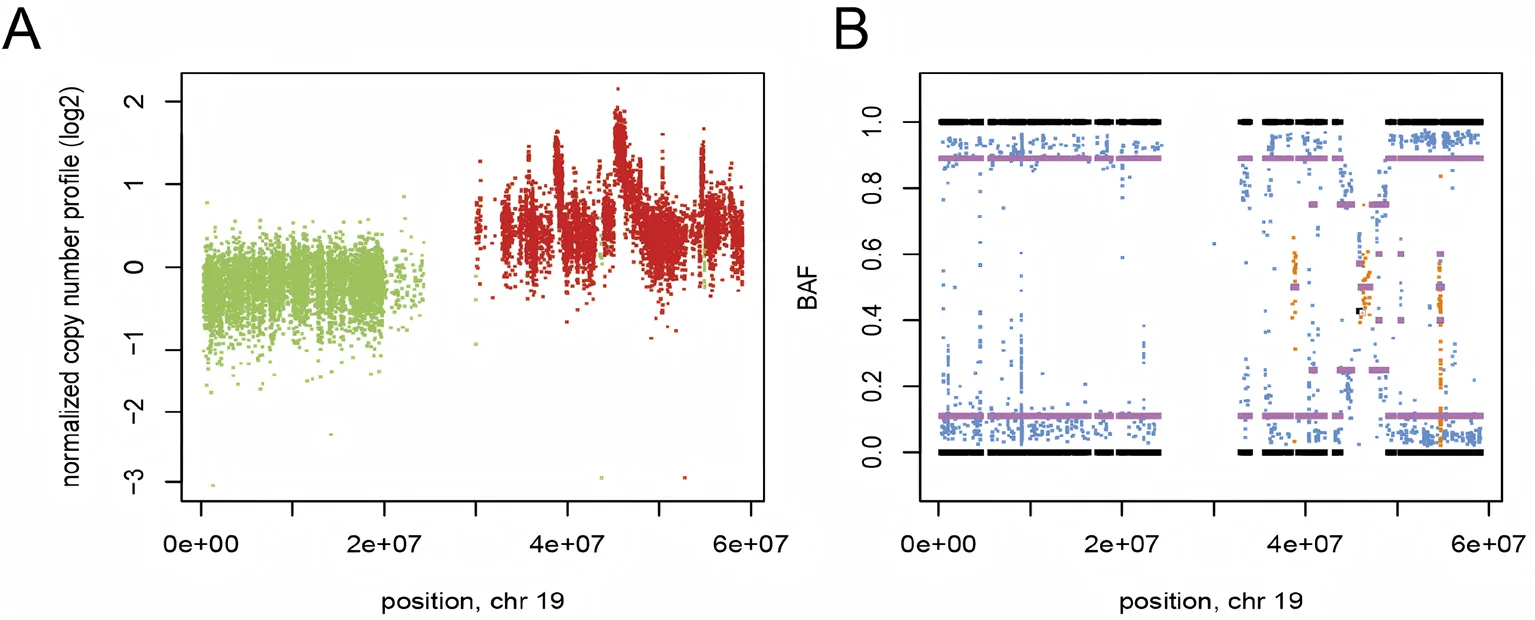
Normalized copy number **(A)** and B-allele frequency [BAF, **(B)**] profiles of chromosome 19. The region harboring SMARCA4 (19p13.2) retains a diploid copy number (Log2 ratio ≈ 0) and a heterozygous BAF pattern without loss of heterozygosity (LOH). These results rule out copy number variations or LOH as a cause of SMARCA4 protein loss, supporting epigenetic silencing as the most likely mechanism.

Adjuvant tislelizumab monotherapy was recommended but self-discontinued by the patient after two cycles for personal reasons. At 23 months, the patient developed intravesical recurrence. Based on HER2 expression (IHC 2+), treatment was switched to disitamab vedotin (2.0 mg/kg) plus tislelizumab (200 mg), both given every 3 weeks (q3w). After three cycles, the patient again discontinued treatment. At 35 months after diagnosis, CT imaging showed progression of tumor invasion into the right ureter compared with 23 months, with worsening hematuria; maximal TURBT was then performed, followed by 8 cycles of disitamab vedotin plus tislelizumab. Subsequent MRI showed progression with invasion of the right pubic bone, which was managed with bilateral internal iliac and vesical artery embolization for palliation. At 48 months, CT showed that the degree of tumor invasion into the right ureter was comparable to that at 35 months (**Figure 3**). After the above comprehensive treatments, the patient’s disease was stable disease (SD).

**Figure 3:**
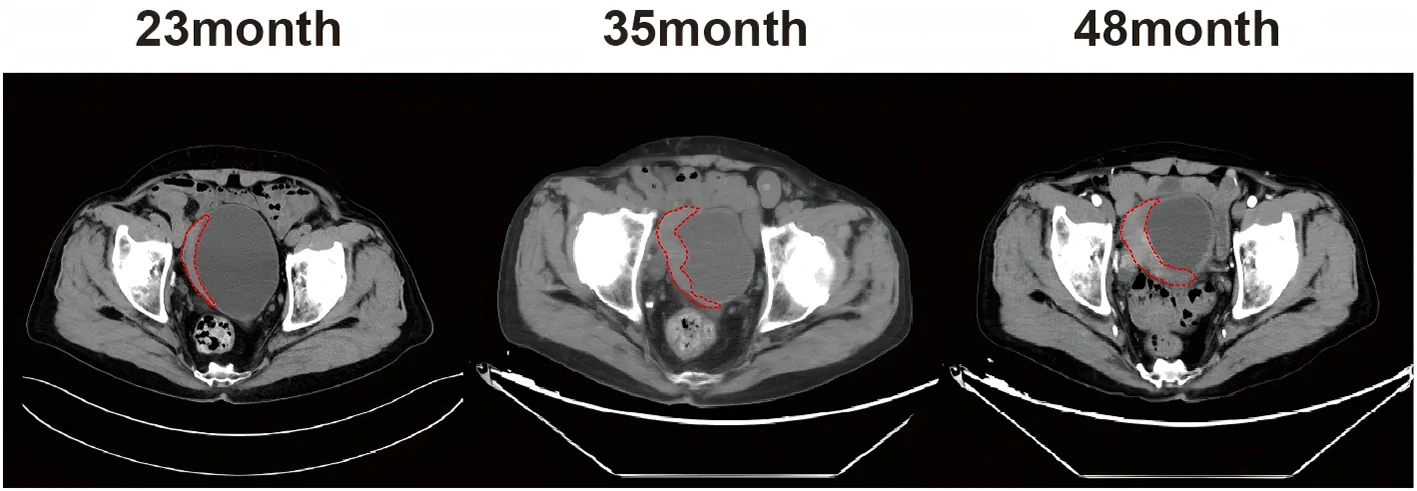
Serial axial contrast-enhanced CT images of the pelvis acquired at the same axial level at 23, 35, and 48 months after diagnosis. The described extent of right ureteric involvement increased between 23 and 35 months and was similar at 35 and 48 months. This local comparison should not be interpreted as a formal overall RECIST 1.1 response assessment.

At the last follow-up, 48 months after diagnosis, the patient was alive with disease and had experienced no grade ≥3 treatment-related adverse events. This is the longest survival reported for this entity, achieved despite repeated therapy interruptions and irregular follow-up.

In summary, the patient was diagnosed in July 2022, underwent partial cystectomy, and received two cycles of adjuvant tislelizumab. Intravesical recurrence occurred at 23 months, prompting combination therapy with disitamab vedotin plus tislelizumab, which was interrupted after three cycles. At 35 months after diagnosis, following TURBT for progressive hematuria, disitamab vedotin plus tislelizumab was resumed for eight cycles. Subsequent bone progression was managed with palliative arterial embolization. At 48 months since diagnosis, the patient remains alive with stable disease.

## 3 Discussion

### 3.1 Clinicopathological spectrum of *SMARCA4*-deficient bladder tumors

*SMARCA4-*deficient tumors arising in the bladder are extremely rare but highly aggressive, with a strong propensity for recurrence and metastasis. **Table 1** summarizes the five cases reported to date, comprising the present case (Case 1) and four previously published cases. All patients were male (age range 48–73 years). *SMARCA4* genetic alterations were identified in only two of the three cases in which sequencing was performed: a nonsense mutation (p.K867*) and a splice-site variant (c.3216-2A>G); the present case was sequencing-negative, and no genomic analysis was undertaken in the remaining two. Distant metastases occurred in four cases, and one patient died of disease five months after diagnosis. Notably, among the five reported cases, the two longest survivors are our patient (48 months) and the nivolumab-treated case reported by Arihara et al. (2). However, that case had high PD-L1 expression, whereas our patient achieved disease control despite low PD-L1 expression. This distinction suggests that *SMARCA4* deficiency may drive an immune-permissive state independently of PD-L1 status, though PD-L1 expression may still contribute to response on an individual basis. Although the number of cases is far too small for any inference about comparative efficacy, this pattern is at least consistent with the hypothesis that checkpoint inhibition, rather than cytotoxic chemotherapy, is the more promising backbone in this subtype.

**Table 1:** Clinicopathological characteristics of five cases of bladder SMARCA4-deficient tumors.

Case	Age/sex	Site & morphology	pTNM	*SMARCA4* genomic finding	Treatment	Thoracic/pulmonary findings	Metastasis/outcome
1	69/M Present study	Right lateral wall diverticulum; large epithelioid/rhabdoid cells	pT3N0	No detectable coding mutation; no CNV or LOH (WES, paired)	Partial cystectomy → tislelizumab ×2 → disitamab vedotin + tislelizumab → TURBT → arterial embolization	No pulmonary or mediastinal lesions on baseline or serial chest CT	Right pubic bone at 45 mo/alive at 47 mo
2	68/M (8)	Left bladder wall and left ureter; rhabdoid-like undifferentiated	pT3N0	p.K867* nonsense (novel)	Surgery + intravesical chemotherapy + toripalimab	Not reported	Sigmoid colon at 16 mo/alive
3	60/M (2)	Bladder (site NA); small-cell rhabdoid with scattered giant cells	NA	c.3216-2A>G splice variant	TURBT + nivolumab (after recurrence)	Not reported	Retroperitoneum, shrinkage after treatment/alive
4	48/M (9)	Right lateral and posterior wall; morphology NA	pT4N1	Not performed	Cystoprostatectomy + adjuvant chemotherapy	Not reported	Lung/died of disease at 5 mo
5	73/M (10)	Left lateral wall; basophilic medium-to-large cells	pT1N0	Not performed	Neoadjuvant GC + toripalimab → elective radical cystectomy	Not reported	No metastasis on imaging/alive

NA, not available or not described; CNV, copy number variation; LOH, loss of heterozygosity; WES, whole-exome sequencing; GC, gemcitabine plus cisplatin; TURBT, transurethral resection of bladder tumor; CT, computed tomography.

### 3.2 Discordance between BRG1 protein loss and the *SMARCA4* genotype

BRG1, the product of *SMARCA4*, is one of the two mutually exclusive ATPase subunits of the SWI/SNF complex. It contains a bromodomain and a helicase/ATPase module, and the energy released by ATP hydrolysis drives nucleosome sliding, eviction, and histone dimer exchange, thereby converting compacted chromatin into an accessible conformation (3). *SMARCA4* acts as a tumor suppressor and is mutated across a wide range of cancers, including ovarian, thoracic, bladder, and colorectal tumors (11). Notably, 35% of tumors with BRG1 loss lack detectable *SMARCA4* mutations (12), and loss of SWI/SNF subunit expression is a recurrent event in undifferentiated and dedifferentiated urothelial carcinoma (12).

Consistent with this, the WHO Classification of Thoracic Tumours regards complete loss of SMARCA4 protein on IHC as sufficient for the diagnosis of a SMARCA4-deficient tumor and does not require confirmatory sequencing, although that recommendation was formulated for thoracic neoplasms and its extrapolation to the urinary tract has not been formally validated (13).

In our case the discordance was more complete than in previously reported tumors, because deletion and LOH were excluded in addition to sequence variants. An epigenetic mechanism is therefore the most plausible explanation. Concordant down-regulation of BRG1 and BRM in tumor cell lines has been attributed to promoter mutation and promoter hypermethylation (14), and both miR-21 and additional microRNAs directly target *SMARCA4* transcripts (15, 16). *SMARCA4* deficiency, however it arises, is closely associated with tumor progression and adverse prognosis (17). From a practical standpoint, the corollary is that a negative sequencing result must never be used to exclude a *SMARCA4*-deficient tumor when IHC shows unequivocal loss with a preserved internal control.

We acknowledge that whole-exome sequencing cannot definitively exclude promoter variants, deep intronic variants, balanced or complex structural rearrangements, or low-frequency subclonal alterations. Therefore, our findings should be interpreted as the absence of detectable coding alterations rather than the complete exclusion of all possible genomic mechanisms. The tumor showed focal and weak expression of CK7, CK20, and P63, which might suggest an epithelial origin; however, undifferentiated malignant tumors can also express keratins. Given the strong expression of stemness markers (CD34 and SOX2), together with the characteristic loss of SMARCA4 (BRG1), the diagnosis of an undifferentiated malignant tumor is favored over carcinoma.

### 3.3 Therapeutic vulnerabilities of *SMARCA4*-deficient tumors

Therapeutic strategies for SWI/SNF-deficient tumors have focused on three synthetic-lethal vulnerabilities: epigenetic, metabolic, and immunologic. Epigenetically, SWI/SNF loss creates EZH2 dependency; EZH2 inhibitors have shown activity in *SMARCA4*-deficient tumors, though toxicity and secondary malignancies limit their use (18–20). Metabolically, *SMARCA4*-deficient cells shift to oxidative phosphorylation and become dependent on glutamine import, a vulnerability exploitable by alanine supplementation combined with complex I inhibition in preclinical models (21). Immunologically, the evidence is more nuanced. Although *SMARCA4* loss may impair antitumor immunity (22), durable responses to PD-1 blockade have been documented in both thoracic and bladder *SMARCA4*-deficient tumors (2, 23, 24). The likely reconciliation is that *SMARCA4* deficiency reduces the probability of response without abolishing it, and conventional biomarkers such as PD-L1 perform poorly in this subtype and should not alone guide treatment decisions.

Beyond these three established vulnerabilities, emerging targeted approaches are being explored. *SMARCA4* loss has been linked to CDK4/6 pathway dependency, and preclinical studies have shown sensitivity to CDK4/6 inhibitors such as palbociclib and abemaciclib (25). Another approach exploits the synthetic lethal relationship between *SMARCA4* and its paralog *SMARCA2*. Since *SMARCA4*-deficient cancers depend on *SMARCA2* for survival, selective *SMARCA2* degraders have been developed (26). PRT3789, a first-in-human *SMARCA2*-selective degrader, has shown robust antitumor activity in *SMARCA4*-deficient models and preliminary clinical activity in patients with *SMARCA4*-mutated cancers (26). These emerging strategies warrant further investigation in *SMARCA4*-deficient bladder tumors, including the subtype reported here.

### 3.4 Interpretation of the present case

Our patient obtained prolonged disease control despite low PD-L1 expression. The mechanism underlying this observation is supported by emerging evidence. In *SMARCA4*-deficient cancers, loss of SWI/SNF complex function leads to an immune-permissive tumor microenvironment characterized by enhanced recruitment of effector immune cells and functionally impaired regulatory T cells (Tregs) (27, 28). These alterations, combined with dysregulated interferon-γ–related gene transcription, create a state of immune engagement that is not adequately reflected by PD-L1 immunohistochemical scoring. Consistent with this, our case exhibited dense lymphoid infiltration within the tumor, which likely contributed to the sustained response to tislelizumab. These findings reinforce that PD-L1 expression alone should not be used to exclude patients with this rare subtype from immunotherapy. Additionally, HER2 expression provided a second actionable target; disitamab vedotin, the HER2-directed ADC used in this case, releases a cytotoxic payload and induces immunogenic tumor cell death, offering a mechanistic rationale for combining this agent with PD-1 blockade. The contribution of each component cannot be separated in a single patient, and the sequence used here—checkpoint inhibitor first, combination after recurrence—was determined by the clinical course rather than by design. Furthermore, the initial two cycles of adjuvant tislelizumab alone are insufficient to permit a definitive conclusion regarding the efficacy of anti-PD1 monotherapy. Recent evidence supports the use of disitamab vedotin combined with immunotherapy in HER2-expressing advanced urothelial carcinoma. The phase 3 RC48-C016 trial demonstrated that disitamab vedotin plus toripalimab significantly improved both progression-free survival (13.1 vs. 6.5 months; HR 0.36) and overall survival (31.5 vs. 16.9 months; HR 0.54) compared with chemotherapy in patients with HER2-expressing (IHC 1+/2+/3+) locally advanced or metastatic urothelial carcinoma, regardless of cisplatin eligibility (29). The phase Ib/II RC48-C014 trial reported an objective response rate of 73.2% and a median overall survival of 33.1 months with the same combination, with benefits observed across HER2 and PD-L1 subgroups (30). Real-world data also support the efficacy of disitamab vedotin combined with tislelizumab in this setting (31). Our patient received disitamab vedotin plus tislelizumab, a regimen consistent with these reported combinations. Although no definitive conclusion can be drawn from a single case, the prolonged survival observed here aligns with the benefit signal reported in these studies.

However, the prolonged survival must be interpreted with caution. The patient received only two cycles of adjuvant tislelizumab before discontinuing treatment on his own. Intravesical recurrence occurred at 23 months, after which he received only three cycles of disitamab vedotin plus tislelizumab before stopping again. A second recurrence occurred at 35 months. Following TURBT, he received eight further cycles of disitamab vedotin plus tislelizumab, along with selective arterial embolization. Given these repeated interruptions and the concurrent local interventions—partial cystectomy, maximal TURBT, and arterial embolization—the relative contributions of systemic therapy versus local procedures cannot be distinguished in a single patient. Intrinsic tumor biology may also play a role. The durable survival observed here should therefore be regarded as the cumulative result of multimodal management rather than evidence for any single component.

### 3.5 Limitations

Several limitations should be acknowledged. First, no causal inference about treatment efficacy can be drawn from a single case, particularly given repeated patient-initiated therapy interruptions. Second, the proposed epigenetic mechanism remains inferential, as promoter methylation and transcript-level analyses were not performed. Third, HER2 status was assessed by IHC alone without *in situ* hybridization confirmation. Finally, comparison with prior cases is limited by heterogeneous reporting and incomplete genomic data.

## 4 Methods

Whole-exome sequencing (WES) was performed on paired tumor and matched normal samples using an Illumina paired-end 150 bp platform. The tumor sample achieved a mean target coverage depth of 211.82×, with >99% of target regions covered at ≥10×. Reads were aligned to hg19 using BWA-MEM. Somatic variants were called using GATK Mutect2, and structural variants and copy-number variations were analyzed using DELLY and Control-FREEC, respectively. Variants were annotated with ANNOVAR against multiple databases (1000 Genomes, COSMIC, ClinVar, dbSNP, ExAC, and gnomAD). The target region spanned 41.7 Mb, and approximately 200 coding somatic mutations were identified. TMB was calculated as the number of coding somatic mutations divided by the target region length, yielding approximately 4.8 mutations/Mb. MSI status was inferred from the WES data; the tumor was classified as microsatellite stable (MSS) based on the low TMB and the small number of frameshift mutations. No pathogenic coding SMARCA4 point mutations or indels were identified, and no deletion or loss of heterozygosity (LOH) was detected at 19p13.2.

Immunohistochemistry (IHC) was performed using the Celnovte CNT300 automated staining platform. The following primary antibodies were utilized: BRG1 (clone 240172F4, Proteintech, 1:100), PD-L1 (clone 2B11D11, Proteintech, 1:200), SMARCB1/INI1 (clone D8M1X, Cell Signaling Technology, #91735, 1:100), SMARCA2/BRM (clone D9E8B, Cell Signaling Technology, #11966, 1:100), CD34 (AF20113, AiFang Biological, 1:200), and SOX2 (AF20094, AiFang Biological, 1:200). PD-L1 expression was evaluated by two independent pathologists using the tumor cell score (TC) and immune cell population score (ICP), with TC <25% and ICP <1% considered low expression. For BRG1, positive staining in intact internal control cells (e.g., stromal cells) was required to validate the assay, and loss of expression was defined as the complete absence of nuclear staining within the tumor cells.

## 5 Conclusions

*SMARCA4*-deficient undifferentiated malignant tumor of the urinary bladder is rare, aggressive, and chemoresistant. This case demonstrates that complete BRG1 loss can occur without detectable *SMARCA4* mutation, copy-number change, or LOH, underscoring that IHC—not sequencing—should be the primary diagnostic test when internal controls are preserved. Clinically, PD-1 blockade combined with a HER-2-directed ADC achieved the longest survival reported for this tumor despite low PD-L1 expression and poor adherence, suggesting that PD-L1 status alone should not preclude immunotherapy. Prospective evaluation of these strategies, alongside epigenetic and metabolic vulnerabilities created by SWI/SNF loss, will require multicenter collaboration and a shared registry for these rare tumors.

## 6 Patient perspective

The patient reported that he twice discontinued treatment against medical advice, which was followed by tumor recurrence. After the second recurrence, he fully adhered to the prescribed regimen, and the disease has since remained stable. He is now alive with disease at 48 months since diagnosis and emphasizes that consistent treatment truly matters.

## Data Availability

The datasets presented in this article are not readily available because they contain identifiable information from a human subject that cannot be shared for legal reasons. The raw sequencing data are available from the corresponding author upon reasonable request for academic purposes only.

## References

[1] Aydogdu C Bukavina L Cheng L Al-Ahmadie HA Alhalabi O Brown JR . Histological subtypes and divergent differentiation of urothelial carcinoma: Histology, genomics, and management implications. Eur Urol. (2026). doi: 10.1016/j.eururo.2026.05.030 42362411 PMC13336308

[2] Arihara Y Omori G Kobayashi K Sugita S Murase K Kubo T . Marked response to nivolumab by a patient with Smarca4‐deficient undifferentiated urothelial carcinoma showing high Pd‐L1 expression: A case report. Cancer Rep(Hoboken). (2024) 7:e2127. doi: 10.1002/cnr2.2127 38923369 PMC11194675

[3] Field NR Dickson K-A Nassif NT Marsh DJ . Smarca4 and Smarca2 co-deficiency: An uncommon molecular signature defining a subset of rare, aggressive and undifferentiated malignancies associated with defective chromatin remodeling. Cancer Lett. (2024) 605:217282. doi: 10.1016/j.canlet.2024.217282 39369768

[4] Li X Tian S Shi H Ta N Ni X Bai C . The golden key to open mystery boxes of Smarca4-deficient undifferentiated thoracic tumor: Focusing immunotherapy, tumor microenvironment and epigenetic regulation. Cancer Gene Ther. (2024) 31:687–97. doi: 10.1038/s41417-024-00732-4 38347129 PMC11101339

[5] Cui M Lemmon K Jin Z Uboha NV . Esophageal carcinoma with *Smarca4* mutation: Unique diagnostic challenges. Pathol Res Pract. (2023) 248:154692. doi: 10.1016/j.prp.2023.154692 37459679

[6] Longo V Catino A Montrone M Montagna ES Pesola F Marech I . Treatment of thoracic Smarca4-deficient undifferentiated tumors: Where we are and where we will go. Int J Mol Sci. (2024) 25:3237. doi: 10.3390/ijms25063237 38542211 PMC10970296

[7] Jiang J Chen Z Gong J Han N Lu H . Thoracic Smarca4-deficient undifferentiated tumor. Discov Oncol. (2023) 14:51. doi: 10.1007/s12672-023-00639-w 37115343 PMC10147882

[8] Lu X Ning C Gao B . A novel oncogenic nonsense mutation of Smarca4 and genetic characteristic analysis of Smarca4 (Brg1)-deficient undifferentiated tumor of the bladder. Virchows Arch. (2026). doi: 10.1007/s00428-026-04402-8 41555058

[9] Richards S Aziz N Bale S Bick D Das S Gastier-Foster J . Standards and guidelines for the interpretation of sequence variants: A joint consensus recommendation of the American College of Medical Genetics and Genomics and the Association for Molecular Pathology. Genet Med. (2015) 17:405–24. doi: 10.1038/gim.2015.30 25741868 PMC4544753

[10] Agaimy A Bertz S Cheng L Hes O Junker K Keck B . Loss of expression of the Swi/Snf complex is a frequent event in undifferentiated/dedifferentiated urothelial carcinoma of the urinary tract. Virchows Arch. (2016) 469:321–30. doi: 10.1007/s00428-016-1977-y 27339451

[11] Ye W An D Ou W-B . Smarca4: Promises and challenges in the treatment of cancers. Cancer Lett. (2025) 625:217811. doi: 10.1016/j.canlet.2025.217811 40398707

[12] Shi M Pang L Zhou H Mo S Sheng J Zhang Y . Rare Smarca4-deficient thoracic tumor: Insights into molecular characterization and optimal therapeutics methods. Lung Cancer. (2024) 192:107818. doi: 10.1016/j.lungcan.2024.107818 38763102

[13] WHO Classification of Tumours Editorial Board . Thoracic Tumours. Lyon: IRAC Press (2021).

[14] Reisman DN Strobeck MW Betz BL Sciariotta J Funkhouser W Jr. Murchardt C . Concomitant down-regulation of Brm and Brg1 in human tumor cell lines: Differential effects on Rb-mediated growth arrest vs Cd44 expression. Oncogene. (2002) 21:1196–207. doi: 10.1038/sj.onc.1205188 11850839

[15] Schramedei K Mörbt N Pfeifer G Läuter J Rosolowski M Tomm JM . Microrna-21 targets tumor suppressor genes Anp32a and Smarca4. Oncogene. (2011) 30:2975–85. doi: 10.1038/onc.2011.15 21317927 PMC3134876

[16] Coira IF Rufino-Palomares EE Romero OA Peinado P Metheetrairut C Boyero-Corral L . Expression inactivation of Smarca4 by micrornas in lung tumors. Hum Mol Genet. (2015) 24:1400–9. doi: 10.1093/hmg/ddu554 25355421 PMC4321447

[17] Fukumoto T Magno E Zhang R . Swi/Snf complexes in ovarian cancer: Mechanistic insights and therapeutic implications. Mol Cancer Res. (2018) 16:1819–25. doi: 10.1158/1541-7786.Mcr-18-0368 30037854 PMC6279546

[18] Duan R Du W Guo W . Ezh2: A novel target for cancer treatment. J Hematol Oncol. (2020) 13:104. doi: 10.1186/s13045-020-00937-8 32723346 PMC7385862

[19] Tischkowitz M Huang S Banerjee S Hague J Hendricks WPD Huntsman DG . Small-cell carcinoma of the ovary, hypercalcemic type–genetics, new treatment targets, and current management guidelines. Clin Cancer Res. (2020) 26:3908–17. doi: 10.1158/1078-0432.Ccr-19-3797 32156746 PMC7415570

[20] Hsu C Konner JA Gounder MM . Epigenetic therapy in a rare ovarian cancer - a double-edged sword. N Engl J Med. (2024) 391:770–2. doi: 10.1056/NEJMc2407971 39167815

[21] Zhu X Fu Z Chen SY Ong D Aceto G Ho R . Alanine supplementation exploits glutamine dependency induced by Smarca4/2-loss. Nat Commun. (2023) 14:1734. doi: 10.1038/s41467-023-38594-3 37210563 PMC10199906

[22] Wang Y Meraz IM Qudratullah M Kotagiri S Han Y Xi Y . Mutation of Smarca4 induces cancer cell-intrinsic defects in the enhancer landscape and resistance to immunotherapy. Cancer Res. (2025) 85:1997–2013. doi: 10.1158/0008-5472.Can-24-2054 40080526 PMC12127800

[23] Henon C Blay JY Massard C Mir O Bahleda R Dumont S . Long lasting major response to pembrolizumab in a thoracic malignant rhabdoid-like Smarca4-deficient tumor. Ann Oncol. (2019) 30:1401–3. doi: 10.1093/annonc/mdz160 31114851

[24] Dong J Zhang J Pan H Wang C Sheng J . Smarca4-deficient Nsclc treated with first-line tislelizumab and fruquintinib achieved remarkable tumor regression: Case report and literature review. Front Immunol. (2025) 16:1521828. doi: 10.3389/fimmu.2025.1521828 40313929 PMC12043563

[25] Tokat ÜM Bilgiç ŞN Adibi A Aydın E Tutar O Cansız H . Cdk4/6 inhibition with dual immunotherapy in chemorefractory Smarca4-deficient undifferentiated tumor: A case report. Front Immunol. (2026) 17. doi: 10.3389/fimmu.2026.1858358 42238573 PMC13226164

[26] Hulse M Wang M Xu C Carter J Agarwal A Lu L . Prt3789 is a first-in-human Smarca2-selective degrader that induces synthetic lethality in Smarca4-mutated cancers. Cancer Res. (2025) 85:1141. doi: 10.1158/0008-5472.CAN-25-1141 40991405

[27] Hu B Lin JZ Yang XB Sang XT . The roles of mutated Swi/Snf complexes in the initiation and development of hepatocellular carcinoma and its regulatory effect on the immune system: A review. Cell Prolif. (2020) 53:e12791. doi: 10.1111/cpr.12791 32162380 PMC7162795

[28] Velut Y Decroix E Blons H Alifano M Leroy K Petitprez F . Smarca4-deficient lung carcinoma is an aggressive tumor highly infiltrated by Foxp3+ cells and neutrophils. Lung Cancer. (2022) 169:13–21. doi: 10.1016/j.lungcan.2022.05.001 35597058

[29] Sheng X Zeng G Zhang C Zhang Q Bian J Niu H . Disitamab vedotin plus toripalimab in Her2-expressing advanced urothelial cancer. N Engl J Med. (2025) 393:2324–37. doi: 10.1056/NEJMoa2511648 41124210

[30] Zhou L Yang KW Zhang S Yan XQ Li SM Xu HY . Disitamab vedotin plus toripalimab in patients with locally advanced or metastatic urothelial carcinoma (Rc48-C014): A phase Ib/II dose-escalation and dose-expansion study. Ann Oncol. (2025) 36:331–9. doi: 10.1016/j.annonc.2024.12.002 39662628

[31] Zhu K Chang Y Zhao D Guo A Cao J Wu C . Expression of Her2 in high-grade urothelial carcinoma based on Chinese expert consensus and the clinical effects of disitamab vedotin-tislelizumab combination therapy in the treatment of advanced patients. Front Pharmacol. (2024) 15. doi: 10.3389/fphar.2024.1355081 38455962 PMC10918465

